# Extensive Prostatic Abscess in an Elderly Patient Requiring Multidisciplinary Drainage

**DOI:** 10.1155/2020/4398561

**Published:** 2020-06-06

**Authors:** John Michael Nesemann, Kathy Huen, Jonathan Bergman

**Affiliations:** ^1^University of California Los Angeles, David Geffen School of Medicine, Los Angeles, USA; ^2^Department of Urology, University of California Los Angeles David Geffen School of Medicine, Los Angeles, USA; ^3^Olive-View UCLA Medical Center, Veterans Association Greater Los Angeles, David Geffen School of Medicine, Los Angeles, USA

## Abstract

*Background.* Prostatic abscess is rare and mainly affects immunocompromised individuals, classically presenting with both systemic and lower urinary tract symptoms. Our case is unique as the patient presented with an exceptionally long duration of symptoms prior to seeing a health-care provider, had no systemic symptoms, and was managed via a multidisciplinary approach. *Case Presentation.* We present a case of a 70-year-old man with type-two diabetes who endured two months of lower urinary tract symptoms and constipation without systemic symptoms prior to seeking medical attention. He had a positive urinalysis and culture and was initially thought to have a urinary tract infection; however, computed tomography scan revealed a large, complex, and multiloculated prostatic abscess. Multidisciplinary drainage of the abscess was performed by interventional radiology and urology. A postoperative Foley catheter was left in place, and the patient recovered without complications. *Discussion.* Prostatic abscess is uncommon and presents almost exclusively in patients with immunocompromising conditions such as diabetes. Prior to the advent of antibiotics, the major causes were gonorrheal and *Staphylococcus aureus* infections, but with the advent of antibiotics, microbial culprits have shifted to gram-negative organisms. Patients typically present with lower urinary tract symptoms, perineal or lower back pain, and systemic symptoms. Management often consists of intravenous antibiotics and surgical drainage either by transrectal ultrasound-guided needle aspiration, or transurethral deroofing of the prostate. Our case highlights the following: (a) the importance of a high index of suspicion for a prostatic abscess in an immunocompromised patient with persistent leukocytosis and perineal pain after treatment with antibiotics and (b) the potential for an early multidisciplinary approach to draining extensive, loculated prostatic abscesses.

## 1. Background

Prostatic abscess is a rare entity, described in 0.5-2.5% of patients presenting with inflammatory prostatitis [1]. Its incidence has declined in current clinical practice and is thought to be due to the introduction of broad spectrum antibiotics and the decreased incidence of gonococcal urethritis [2]. Because of its wide range of nonspecific presenting symptoms, it is commonly misdiagnosed as acute bacterial prostatitis or urogenital chronic pelvic pain syndrome, which are far more common [3]. While classically thought of as a sequela of untreated primary urinary tract infection, it is now a marker of men with immune compromise or chronic medical conditions such as diabetes mellitus (DM), human immunodeficiency virus (HIV), hepatic cirrhosis, renal dialysis, chronic indwelling catheters, genitourinary instrumentation, among others [4]. Prostatic abscess is thought to arise from acute or chronic bacterial prostatitis or to develop de novo from retrograde flow of contaminated urine during micturition or from hematogenous seeding. The microbial profile of prostatic abscess has changed drastically since the preantibiotic era, with a majority of cases now being gram-negative in origin and a rising number involving multidrug-resistant organisms [5]. We describe a case of bacterial prostatic abscess in a diabetic patient who waited an exceptionally long time, i.e., two months, prior to seeking medical attention and lacked systemic symptoms. We emphasize the high index of suspicion necessary to diagnose prostatic abscess and review proposed management guidelines, highlighting the potential for an early multidisciplinary approach to the treatment of extensive prostatic abscesses.

## 2. Case Presentation

A 70-year old man with a history of uncontrolled diabetes (hemoglobin A1c of 16.6%), diabetic foot ulcers for the past three years, and hypertension presented to the emergency room with a two month history of frequency, urgency, nocturia, and constipation requiring manual disimpaction. He denied a history of prostatitis or benign prostatic hyperplasia (BPH); however, he reported a resolved perirectal abscess one year prior to presentation. His lower urinary tract symptoms (LUTSs) had started with difficulty voiding and progressed over two months to frequency and nocturia ten to twelve times per night. He had started wearing a diaper at night and noticed drops of blood in the diaper over the past week but was unsure of the source. Three days prior to presentation, he developed perineal pain radiating to his urethra and rated it as 10/10. His family stated he had been ignoring his symptoms, but he was brought into the emergency department by his wife when she noticed the blood in his diaper. He denied any prior bleeding, fevers, chills, vomiting, or abdominal or flank pain.

On admission, he was afebrile with a heart rate of 95 and a blood pressure of 93/50. His hypotension improved with fluid resuscitation. Physical exam was remarkable for suprapubic tenderness on palpation and an enlarged and mildly tender prostate on digital rectal exam; however, there was no prostatic fluctuance or purulent urethral discharge. A 16 French Foley catheter was placed, and the initial 800 cc of urine was bloody but quickly cleared with irrigation.

Labs were remarkable for leukocytosis of 17.6/mm3, hyperkalemia of 7 mEq/L, bicarbonate of 18 mEq/L, anion gap of 18 mEq/L, creatinine of 1.46 mg/dL, BUN of 36 mg/dL, and a glucose of 883 mg/dL. His urinalysis showed glucose >1000 mg/d, positive ketones, >182 white blood cells/hpf(WBC), negative nitrite, 3+ red blood cells/hpf (RBC), and positive bacteria. Urine culture grew penicillin-resistant, methicillin-sensitive *Staphylococcus aureus* (MSSA), but blood cultures remained negative throughout the hospital stay. He was admitted to the hospital for management of his electrolyte abnormalities. The patient's negative blood cultures made invasive *S. aureus* less likely. As his urine culture grew *S. aureus*, it was suspected he had an isolated urinary tract infection (UTI) and he was started on IV ceftriaxone 1 gram every 24 hours for treatment.

**Figure 1:**
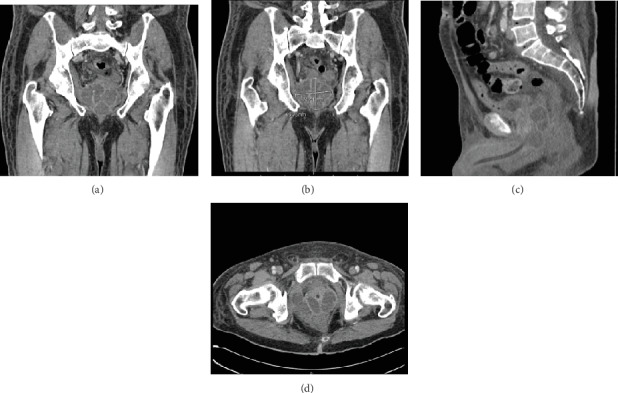
CT of the pelvis with contrast showing the multiloculated prostatic abscess in: (a) coronal view, (b) coronal section with measurements, (c) sagittal view showing involvement of the right perirectal/perianal region, and (d) axial view showing invasion of the right pelvic sidewall/obturator muscle.

The patient's pain and leukocytosis persisted after three days of hospitalization. After discussion with the primary medical team, it was decided there was a wide differential as to the cause of his urinary retention and leukocytosis and an intravenous contrast enhanced pelvic computed tomography (CT) scan was ordered to obtain more information. It demonstrated a complex multiloculated rim-enhancing fluid collection in the pelvis, predominantly centered within the prostate but extending to the right seminal vesicle, right pelvic sidewall, and the perianal region consistent with a prostatic abscess (Figure 1). The abscess measured 7.8 cm × 6.6 cm. Transesophageal echocardiogram revealed no cardiac vegetations. As his urine culture showed sensitivity to methicillin, he was started on 2 grams of cephazolin every 8 hours. After discussion with radiology, it was felt that the patient would benefit from multidisciplinary treatment due to the multiloculated and extensive nature of his abscess. Thus, a combined approach was pursued where interventional radiology (IR) performed a CT-guided percutaneous drainage of the posterior fluid collection, yielding approximately 10 mL of purulent fluid.

Our patient then underwent transurethral deroofing (TUD) of the abscess for which he was placed in the low lithotomy position after a time-out and initiation of anesthesia. He was given 1 g of vancomycin preoperatively and prepped and draped in a sterile fashion. After dilation, a 26-French rigid resectoscope was inserted. The prostate was noted to be about 2 cm in size and no frank pus was noted. The location of the abscess was determined using preoperative CT imaging. Upon identification of the ureteral orifice, we began to shave away thin layers of the prostate from both the right and left lateral walls. Once pus began to escape into the urethral cavity, several thin layers of the prostate were sequentially shaved away to allow for further evacuation of the abscess cavity (Video 1). Throughout this process, the bladder neck and anterior prostate were spared. A total of 70 mL of pus was removed. A final, vigorous digital rectal exam was performed to expel any residual pus. The infected urine was sent for culture, and the prostate chips were also irrigated out.

An 18-French 3-way Foley catheter was left in place after the procedure. Both wound and urine cultures grew penicillin-resistant, methicillin-sensitive *S. aureus*. He was transferred to the intensive care unit (ICU) postoperatively due to his risk of sepsis. His cefazolin regimen was continued, and his leukocytosis resolved. The patient did not experience any adverse events immediately following the intervention and reported tolerating the procedure well.

**Figure 2:**
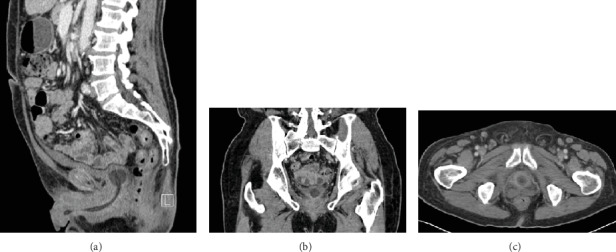
CT of the pelvis: (a) sagittal, (b) coronal, and (c) axial views of the prostate showing near resolution of the abscess with minimal residual fluid seen in the left posterior aspect at the base of the prostate.

He was discharged home where he completed a four-week course of IV cefazolin. He returned to clinic 13 days later, where he was unable to pass a void trial. He passed a repeat void trial 16 days later, and the Foley catheter was removed. Almost a month and a half after the transurethral unroofing of his prostatic abscess, he presented to the emergency department for hyperkalemia and was found to have a post-void residual (PVR) of 400 cc. A Foley was placed and a repeat CT of the abdomen and pelvis showed no hydronephrosis and “near resolution of the previously demonstrated multi-loculated prostatic abscess” (Figure 2). His hyperkalemia resolved, and he was discharged from the medicine service with a Foley two days later. He passed a subsequent void trial, and on telephone follow-up, the patient reported he had no further adverse events and was happy with the outcome of his intervention.

## 3. Discussion and Conclusion

Prostatic abscess is an uncommon disease due to the widespread and early use of antibiotics in most individuals presenting with lower urinary tract symptoms. It most commonly presents in patients with chronic medical conditions such as diabetes (the most common), cirrhosis, renal dialysis, and chronic indwelling catheters [6]. Other risk factors include HIV or other immunocompromising conditions and recent urogenital instrumentation or surgery. Prostatic abscess spreads hematogenously in approximately one quarter of patients, as may be the case with this patient's diabetic foot ulcers, but urinary tract infections and acute bacterial prostatitis are additional etiologies [2].

Prior to the advent of antibiotics, prostatic abscesses were a common complication of gonorrheal infection in otherwise healthy men, with *Staphylococcus aureus* being the other common causative organism [7]. The introduction of antibiotics shifted the microbial culprits to mostly gram-negative organisms, which have been detected in 60-90% of prostatic abscess cultures [8, 9]. Of the gram-negatives, the most common is *Escherichia coli* with 75% of isolates susceptible to first-line antibiotics in the 1990s but only 22% in the 2000s [5]. Recently, there have been increasing numbers of case reports detailing incidences of both methicillin-sensitive and resistant *S. aureus*, with 33 cases reported in the literature as of 2014 [10]. Other reported bacterial pathogens include *Klebsiella pneumoniae*, *Pseudomonas aeruginosa*, *Enterococcus* species, *Streptococcus* species, and *Burkholderia pseudomallei* [2, 5].

Prostatic abscess classically occurs in middle-aged men who present with hesitancy, weakened stream, dysuria, fever, perineal discomfort or low back pain, and leukocytosis. Urinary retention occurs in up to one-third of patients [2]. This case is atypical in that the patient had an exceptionally long duration of symptoms prior to presenting for care. Moreover, he denied systemic symptoms, which are the only presenting symptom in up to one-third of patients [2], and was afebrile, which according to Weinberger et al.'s [8] report, fever occurs in up to 72% of cases. A tender prostate is typically present on physical exam, but a normal digital rectal exam has been reported in 23% of patients [9]. While fluctuance is pathognomonic, its incidence varies from 16 to 88% [4, 8]. As symptoms are variable, diagnosing the condition requires a high index of suspicion in any patients with risk factors who present with lower urinary tract symptoms and signs of infection. When clinically suspected based on systemic and lower urinary tract symptoms in a patient with DM or other immunosuppressive conditions, the workup for prostatic abscess includes imaging with computed tomography or transrectal ultrasonography, and blood and urine cultures.

There is no clear consensus on treatment, but management typically consists of a combination of oral or parenteral antibiotics with surgical drainage via either transrectal ultrasound- (TRUS-) guided drainage or transurethral deroofing, depending on the size and location of the abscess [4, 11, 12]. As Collado et al. [9] reported symptoms of sepsis in 19% of their patients, it can be assumed that prostatic abscesses are high risk for sepsis and should be considered for inpatient management. These patients should be given parenteral antibiotics, with possible options including third-generation cephalosporins or aminoglycosides with ampicillin. Stable patients with a small abscess (<1 cm) may be tried on conservative therapy with serial imaging to ensure resolution; however, most individuals require surgical drainage [11, 13].

The most common surgical drainage option is TRUS-guided needle aspiration, which can be carried out at the bedside and allows for diagnosis and treatment in a single procedure. It has a low rate of infectious complications but serious, and sometimes fatal, episodes of sepsis have been reported [5]. It is also preferred for hemodynamically unstable patients as the anesthesia required for operating on these patients carries a high risk of further deterioration. However, the greatest limitation of this mode of treatment is abscess recurrence or incomplete treatment, which ranges from 16 to 22% [14, 15]. Some experts advocate transurethral deroofing (TUD) of the prostate for large, multiloculated abscesses (as in this patient) as up to one-third of prostatic abscess patients will eventually need transurethral resection of the prostate [5]. This is especially true if the prostate is very large (>80 g), there are residual abscesses, and if the patient suffers from symptomatic benign prostatic hyperplasia [12]. TUD is associated with lower rates of recurrence, as low as 7% in one study [12], and significant shorter hospital length when compared to needle aspiration [14]. A 2017 study of 44 patients with large (>2 cm) or multiple abscesses or abscesses not responding to antibiotic therapy compared patients who had received TRUS-guided needle aspiration with those who had received TUD and found that 96% of patients responded to transurethral deroofing while only 84.1% responded to TRUS-guided needle aspiration (*p* < 0.23) [16]. They also found that six patients in the TRUS group had abscess recurrence while only one in the transurethral deroofing group suffered recurrence (*p* < 0.03). TUD of the prostate carries the same risks as transurethral resection of the prostate, such as retrograde ejaculation, urethral stricture, and incontinence.

In the case of our patient, it was decided to pursue a multidisciplinary approach to management combining TUD of the abscess with a posterior needle aspiration. As our patient had a large (>2 cm), multiloculated abscess, we chose to pursue TUD as this is often recommended for large abscesses [12], and has been associated with lower rates of recurrence and shorter hospital stays [12, 14, 16]. Since the abscess extended out from the prostate to the right seminal vesicle, right pelvic sidewall, and the perianal region, it was decided a TUD of the abscess alone would not adequately drain all of the abscess cavities. Thus, a needle aspiration of the posterior fluid collection was also performed by IR. We believed this would give the patient the best chance at recovering from this serious condition.

The prostate deroofing procedure has been described by other authors [16, 17], and we have described our version in the case presentation. Our procedure is most similar to that described by Goyal et al. [17] as we spared the bladder neck and anterior prostate, although our deroofing was more focused as we did not resect other portions of the prostate once adequate drainage had been achieved. There is no mention of digital rectal exam during surgery in order to expel abscess contents, but we believe this maneuver was helpful.

Many of the case reports in the literature describe attempting TRUS-guided drainage, and if there is no improvement, proceeding to TUD of the prostate [18–21]. Here, we report a description of a multidisciplinary approach to draining an extensive and loculated prostate abscess. We believe this approach may be useful for abscess drainage as it has the potential to access multiple areas of loculated abscesses and thus theoretically provide greater drainage compared to a single approach. While this case report presents a new management possibility, the evidence is limited due to the single patient experience reported and lack of control group for comparison. However, further study could elucidate the potential benefits of this approach to the drainage of extensive prostate abscesses.

This case illustrates the importance of a high index of suspicion for a prostatic abscess if there is persistent leukocytosis and perineal pain in an immunocompromised patient with a urinary tract infection after treatment with intravenous antibiotics. Finally, the TUD- and IR-guided percutaneous drainage performed in quick succession for this patient highlights the potential for an early multidisciplinary approach to draining extensive, loculated prostatic abscesses.

## Data Availability

The authors confirm that the data supporting the findings of this study are available within the article.

## Abbreviations

LUTS: Lower urinary tract symptoms
DM: Diabetes mellitus
HIV: Human immunodeficiency virus
WBC: White blood cells
RBC: Red blood cells
MSSA: Methicillin-sensitive Staphylococcus aureus
UTI: Urinary tract infection
IV: Intra-venous
CT: Computed tomography
ICU: Intensive care unit
PVR: Post void residual
TRUS: Trans-rectal ultrasound
TUD: Trans-urethral deroofing
IR: Interventional radiology.

## Supplementary Materials

Supplementary materials Video 1: This video demonstrates shaving away thin layers of the prostate. Prior to the start of the video the urethral orifice was identified and we began to shave away thin layers of the prostate from the lateral walls. As shown in the video, once pus began to escape into the urethral cavity several more layers of the prostate were sequentially shaved away to allow for further evacuation of the abscess cavity. Throughout this process the bladder neck and anterior prostate were spared.

## References

[1] Langer J. E., Cornud F. (2006). Inflammatory disorders of the prostate and the distal genital tract. *Radiologic Clinics of North America*.

[2] Ackerman A. L., Parameshwar P. S., Anger J. T. (2018). Diagnosis and treatment of patients with prostatic abscess in the post-antibiotic era. *International Journal of Urology*.

[3] Nickel J. C., Downey J., Hunter D., Clark J. (2001). Prevalence of prostatitis-like symptoms in a population based study using the National Institutes of Health chronic prostatitis symptom index. *The Journal of Urology*.

[4] Granados E. A., Riley G., Salvador J., Vicente J. (1992). Prostatic abscess: diagnosis and treatment. *The Journal of Urology*.

[5] Bhagat S. K., Kekre N. S., Gopalakrishnan G., Balaji V., Mathews M. S. (2008). Changing profile of prostatic abscess. *International Braz J Urol*.

[6] Naboush A., Yassine A. A., Yasmin M., Mobarakai N. (2013). Community-acquired methicillin-resistant Staphylococcus aureus prostatic abscess presenting as acute urinary retention: a case report and review of the literature. *Case Reports in Infectious Diseases*.

[7] Sargent J. C., Irwin R. (1931). Prostatic abscess: a clinical study of 42 cases. *The American Journal of Surgery.*.

[8] Weinberger M., Cytron S., Servadio C., Block C., Rosenfeld J. B., Pitlik S. D. (1988). Prostatic abscess in the antibiotic era. *Reviews of Infectious Diseases*.

[9] Collado A., Palou J., Garcia-Penit J., Salvador J., de la Torre P., Vicente J. (1999). Ultrasound-guided needle aspiration in prostatic abscess. *Urology*.

[10] Jana T., Machicado J. D., Davogustto G. E., Pan J. J. (2014). Methicillin-resistant Staphylococcus aureus prostatic abscess in a liver transplant recipient. *Case Reports in Transplantation*.

[11] Abdelmoteleb H., Rashed F., Hawary A. (2017). Management of prostate abscess in the absence of guidelines. *International Braz J Urol*.

[12] Elshal A. M., Abdelhalim A., Barakat T. S., Shaaban A. A., Nabeeh A., Ibrahiem E.-H. (2019). Prostatic abscess: objective assessment of the treatment approach in the absence of guidelines. *Arab Journal of Urology*.

[13] Ludwig M., Schroeder-Printzen I., Schiefer H. G., Weidner W. (1999). Diagnosis and therapeutic management of 18 patients with prostatic abscess. *Urology*.

[14] Jang K., Lee D. H., Lee S. H., Chung B. H. (2012). Treatment of prostatic abscess: case collection and comparison of treatment methods. *Korean Journal of Urology*.

[15] Gogus C., Ozden E., Karaboga R., Yagci C. (2004). The value of transrectal ultrasound guided needle aspiration in treatment of prostatic abscess. *European Journal of Radiology*.

[16] Purkait B., Kumar M., Sokhal A. K., Bansal A., Sankhwar S. N., Bhaskar V. (2019). Outcome analysis of transrectal ultrasonography guided aspiration versus transurethral resection of prostatic abscess: 10 years’ experience from a tertiary care hospital. *Arab Journal of Urology*.

[17] Goyal N. K., Goel A., Sankhwar S., Dalela D. (2013). Transurethral resection of prostate abscess: is it different from conventional transurethral resection for benign prostatic hyperplasia?. *ISRN Urology*.

[18] Docekal J., Hall J., Reese B., Jones J., Ferguson T. (2013). A rare presentation of community acquired methicillin resistant Staphylococcus aureus. *Case Reports in Infectious Diseases*.

[19] Carroll D. E., Marr I., Huang G. K. L., Holt D. C., Tong S. Y. C., Boutlis C. S. (2017). Staphylococcus aureus prostatic abscess: a clinical case report and a review of the literature. *BMC Infectious Diseases*.

[20] Park S. C., Lee J. W., Rim J. S. (2011). Prostatic abscess caused by community-acquired methicillin-resistant Staphylococcus aureus. *International Journal of Urology*.

[21] Lachant D. J., Apostolakos M., Pietropaoli A. (2013). Methicillin resistant Staphylococcus aureus prostatic abscess with bacteremia. *Case Reports in Infectious Diseases*.

